# Detection and attribution of urbanization effect on flood extremes using nonstationary flood‐frequency models

**DOI:** 10.1002/2015WR017065

**Published:** 2015-06-16

**Authors:** I. Prosdocimi, T. R. Kjeldsen, J. D. Miller

**Affiliations:** ^1^ Centre for Ecology & HydrologyWallingfordUK; ^2^ Department of Architecture and Civil EngineeringUniversity of BathBathUK

**Keywords:** flood‐frequency analysis, nonstationarity, urban cover, change attribution

## Abstract

This study investigates whether long‐term changes in observed series of high flows can be attributed to changes in land use via nonstationary flood‐frequency analyses. A point process characterization of threshold exceedances is used, which allows for direct inclusion of covariates in the model; as well as a nonstationary model for block maxima series. In particular, changes in annual, winter, and summer block maxima and peaks over threshold extracted from gauged instantaneous flows records in two hydrologically similar catchments located in proximity to one another in northern England are investigated. The study catchment is characterized by large increases in urbanization levels in recent decades, while the paired control catchment has remained undeveloped during the study period (1970–2010). To avoid the potential confounding effect of natural variability, a covariate which summarizes key climatological properties is included in the flood‐frequency model. A significant effect of the increasing urbanization levels on high flows is detected, in particular in the summer season. Point process models appear to be superior to block maxima models in their ability to detect the effect of the increase in urbanization levels on high flows.

## Key Points

- Urbanization is found to have an impact on high flows in a urbanized catchment
- The use of point processes is advocated for trend detection and attribution
- The use of process‐related covariates gives a better representation of change

## 1 Introduction

Frequency analysis of extreme flood events is routinely being conducted assuming that the events can be adequately represented by a stationary modeling framework. Hydrologists have nevertheless always been aware that this assumption of stationarity is, at best, a convenient approximation given the constant anthropogenic and natural changes observed in catchments [*Lins and Cohn*, 2011; *Stedinger and Griffis*, 2011]. Traditionally, nonstationarity in flood estimation was either ignored or sometimes acknowledged through the simple use of multiplication factors. For example, design rainfall and flood estimates are routinely increased by a factor between 20% and 30% to account for future impacts of climate change [*Madsen et al*., 2014], similarly urbanization is often accounted for by first deriving flood statistics as if a catchment is rural and then postadjusting the as‐rural estimates according to the level of urbanization in a given catchment [*Kjeldsen*, 2010; *Madsen et al*., 2014].

As *Montanari and Koutsoyiannis* [2014] point out, before switching to a fully nonstationary modeling paradigm, one should provide scientific evidence that changes in the generation of extreme events can be detected. If trends in the extreme processes are detected, the causes of such changes should be investigated, to rule out, as far as possible, the influence of spurious information contained in short and highly variable flood series. Therefore, as *Merz et al*. [2012] point out, next to the detection of trend, rigorous attribution is needed, i.e., an understanding of the drivers of the detected change.

Many investigations have been carried out to detect and potentially attribute changes in high‐flow regimes. A number of studies focus on the changes in time of block maxima, although the effect of other covariate on the properties of the distribution of hydrological extremes has also been explored. See, among others, *Delgado et al*. [2010], *Vogel et al*. [2011], and *Sun et al*. [2014].

The impacts of urbanization on catchment flood characteristics have, at least conceptually, been accepted for several decades [*Leopold*, 1968; *Bailey et al*., 1989; *Packman*, 1980; *Shuster et al*., 2005]. Various studies have investigated whether an increase in the magnitude of observed flow records can effectively be linked to changes in the urbanization levels [e.g., *Beighley and Moglen*, 2002; *Konrad and Booth*, 2002; *Villarini et al*., 2009; *Vogel et al*., 2011]. In a study of AMAX series from 200 urbanized catchments in the UK, *Kjeldsen* [2010] found that L‐CV decreased and L‐SKEW increased with increasing urbanization, though none of these effects were particularly strong. The increase of the magnitude of peak flows in urbanizing catchments is due to a number of factors and the interplay between them. A reduction in the natural infiltration can be expected due to the introduction of impervious surfaces, leading to an increase in the volume of storm runoff. At the same time, the replacement of natural water courses with more efficient man‐made drains reduces the lag‐time of the runoff response (see discussions in, e.g., *Kjeldsen et al*. [2013] and *Miller et al*. [2014]). Next, the connectivity to drainage, termed effective impervious area (EIA) or directly connected impervious area (DCIA), would also play a role in the catchment response to rainfall events [*Shuster et al*., 2005]. The impact of urbanization could then be different according to the perviousness of the catchment before the large increases in urbanization levels, or the design of the new impervious cover. Finally, urbanization is likely to affect the magnitude of smaller, more frequent, floods rather than the really large and rare events [*Hollis*, 1975]. As we consider larger storms, the relative effect of the impervious area decreases as the high intensity and volume of rainfall exceeds infiltration capacity of pervious surfaces, causing the nonurban parts to behave more like an impervious surface.

As discussed in *Prosdocimi et al*. [2014] and later in this work, the record length available for annual maxima series (typically around 35 years in the UK) is not large enough to allow for an unequivocal detection and attribution of trends via statistical testing, and the analysis of such block maxima can be highly influenced by anomalies in the data series. Beside block maxima, peaks‐over‐the threshold series (POT), also known as a Partial Duration Series (PDS), are frequently used to assess the behavior of extreme events [see *Madsen et al*., 1997; *Lang et al*., 1999]. It can be shown that a connection exists between the models typically used to estimate flood frequency using either block maxima or the POT series, and both methods would asymptotically lead to equivalent inference. The performance of different estimation methods applied to block maxima and POT series are discussed in *Madsen et al*. [1997]. The analysis of threshold exceedances would potentially be a better tool to detect and attribute the effect of different variables on the high‐flow properties as this would ensure that a larger number of data points (all characterizing the extremal part of the distribution) are used to investigate the effects of the variables on high flows. Threshold exceedances series would also potentially be less sensitive to outliers and leverage points present in the data. In particular, the point process characterization for threshold exceedances is advocated as this characterization allows for a simpler approach to nonstationarity modeling and can be shown to be equivalent to the classical peaks‐over‐threshold modeling frequently used in hydrology [*Coles*, 2001].

In this work, we present methods for attributing flood change that are in line with the suggestions by *Merz et al*. [2012] within a case‐control framework, by comparing high‐flow series of two very similar catchments in North England, which differ mainly with regard to the spatiotemporal development of urbanization. The case catchment went through significant urbanization over the study period (1976–2010), while the paired land use in the control catchment remained largely unchanged from the 1970s till present times. It is assumed that the behavior of the two nearby catchments is broadly similar (a realistic assumption, as shown by *Andréassian et al*. [2012]), so that changes in the peak flow behavior would reflect the changes in the catchment properties. Further, the potential effects of other important drivers are accounted for in the models, which can explain a large part of the variability observed in the data. Assuming that the drivers included in the models can explain a large part of the natural variability of flow peaks, the detected change in the urbanizing catchment can be attributable solely to the increasing urban cover, in particular when compared to the unchanged patterns in the high flows of the rural paired catchment. Paired catchments have been widely employed in the assessment of the effects of changes in the catchment vegetation on river flow, in particular in forest hydrology [*Brown et al*., 2005; *Alila et al*., 2009]. In this study, the effects of the changes in land use on peak flows are investigated by assessing if any changes can be identified in the observed peak flows of the paired catchments. A possible different approach would be to compare the observed peak flows and the peak flows which one could expect from an hydrological model simulated under a different land use scenario, as in, among others, *Brath et al*. [2006] and *Harrigan et al*. [2014]. Furthermore, in this study a variable which actually describes the dynamic evolution of the catchment land use is used rather than relying on time as a surrogate covariate. This allows for a stronger and more process‐based attribution, so that the attributed impact can be more easily extrapolated for increasing levels of urban cover. Also, rather than relating the increase in the urban extent to the peak flow values only, the estimation focuses on the net effect of urbanization after the climate variability is taken into account, in line with *López and Francés* [2013]. In order to have a better assessment of the potential effects of urbanization on high flows, both annual and seasonal data are analyzed in this work. This allows for a better understanding of the type of changes in floods which might be expected with increasing urbanization levels.

## 2 Case Study Description

To identify the effects of urbanization on catchment flood response, it was necessary to identify a catchment with increasing levels of urban land use and a nearby hydrologically similar rural catchment which experienced no significant change in land use. If, after accounting for natural variability, any significant trends can be detected in the high‐flow data observed in the urbanizing catchment (the case catchment) but not in the data from the rural catchment (the control catchment), these changes could be attributed to the increasing urbanization with a greater degree of confidence.

**Figure 1:**
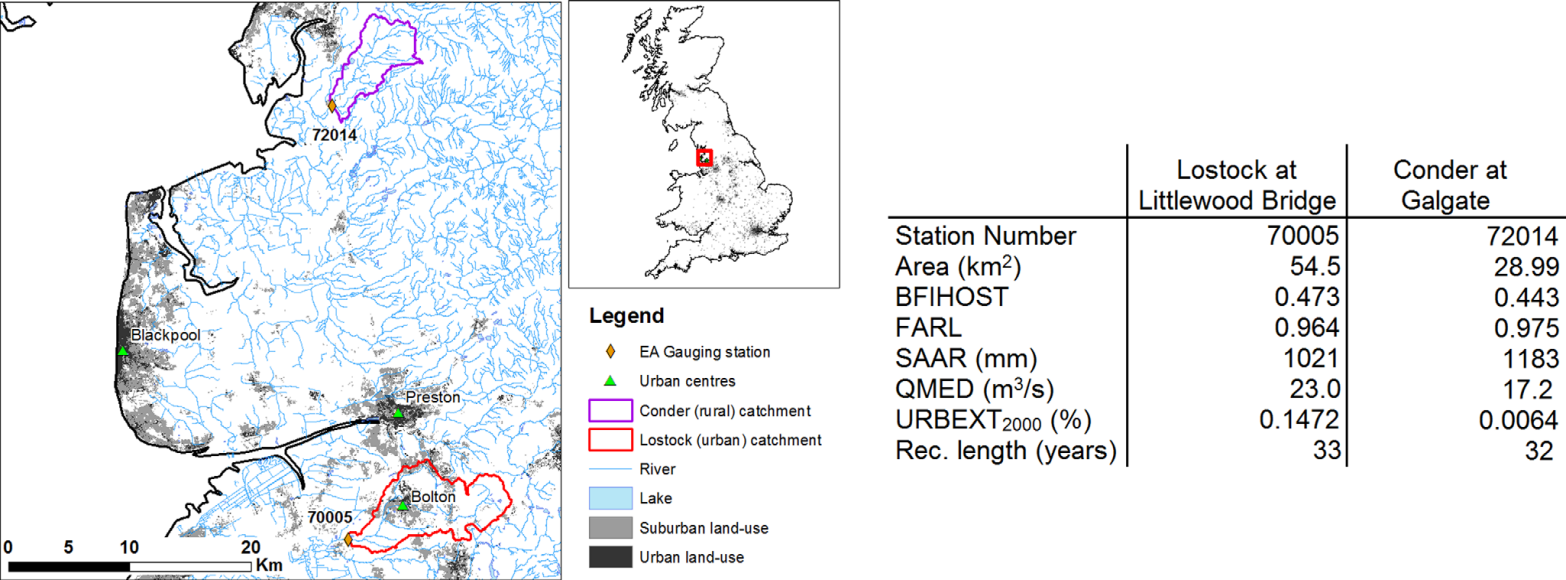
Location of the two study catchments upstream of gauging station 70005 (urbanized catchment) and station 72014 (rural catchment). Key catchment descriptors [from *Institute of Hydrology*, 1999] are also displayed.

Using the catchment similarity measure developed for regional frequency analysis in British catchments [*Environment Agency*, 2008], the urbanized catchment of Lostock at Littlewood Bridge (gauging station 70005) was selected as a case study, while the nearby Conder at Galgate (gauging station 72014) was taken as a control catchment. The two catchments are located in the North West of England (see Figure 1) and have fairly long high‐quality instantaneous flow records.

Key catchment descriptors of the two catchments, taken from *Institute of Hydrology* [1999], are also shown in Figure 1: BFIHOST is a Base Flow Index representative of catchment responsiveness; FARL is an index of Flood Attenuation by Reservoirs and Lakes; SAAR is the Standard period Average Annual Rainfall (1961–1990); QMED is the median annual maximum flow, and URBEXT_2000_ is an index of urban extent in the year 2000. Beside the URBEXT_2000_ values, the other characteristics of the two catchments are quite similar, although the area upstream of Lostock is larger. The Conder at Galgate catchment is a predominantly rural catchment, which has seen very little change in land use, as testified by its inclusion in the undisturbed benchmark catchments used by *Hannaford and Marsh* [2008]. In contrast, the Lostock at Littlewood Bridge catchment experienced a significant increase in urban extent. Urban extent is calculated as a weighted mean of the Urban and Suburban land use classes defined in the Land Cover Map 2000 data set (LCM2000) [*Fuller et al*., 2002].

**Figure 2:**
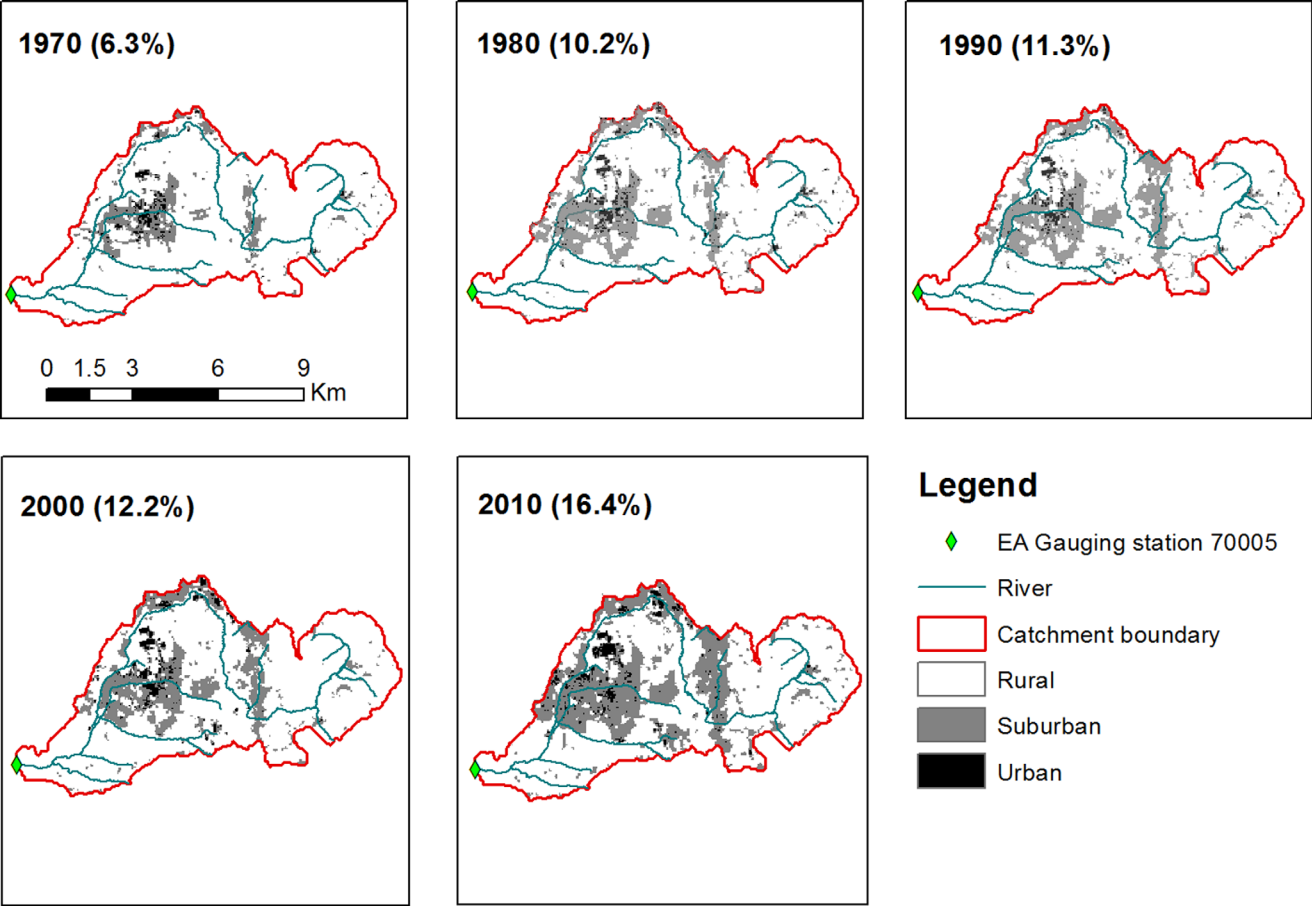
Evolution of the urban extent in the Lostock at Littlewood Bridge catchment (station 70005). The year to which the image refers to is in indicated, with the corresponding URBEXT value in parentheses.

Additionally, in catchment 70005 the land use classes and associated URBEXT value were derived for each decade using the method for mapping historical change in urban land use and impervious cover developed by *Miller and Grebby* [2014]. This involved the processing of digitized historical maps produced by the UK Ordnance Survey to produce mapping of urban land use and has been demonstrated to provide robust estimates of urbanization. However, the values are only point estimates of urban extent for a single decade and cannot provide detailed information on a finer time scale. The urban catchment 70005 (Figure 2) changed from a predominantly rural catchment in 1970 (URBEXT = 6.3%) to one having large areas of urban development in 2010 (URBEXT = 16.4%): a 260% increase in URBEXT.

URBEXT is a relatively simple measure developed in response to the need for a standard method to quantify the artificially impervious cover of a catchment across the whole UK. It is a proxy for the hydrological and hydraulic alteration of a catchment associated with urban development and makes no direct account for the specific physical changes that will have occurred such as increased drainage network density or installation of attenuating features. It is nevertheless a valid indicator of changes in the catchment properties and has the great advantage of being relatively easy to implement for any given catchment across the country.

## 3 Hydrometric and Land Use Data

Instantaneous peak flow data recorded at 15 minute intervals for the stations 70005 and 72014 were acquired from the Environment Agency. A water‐year in the UK runs from 1 October to 30 September: throughout the rest of the paper, all the references to annual and yearly quantities should be interpreted as referring to water‐years, rather than calendar years. The data were checked against the annual maxima published by Hi‐Flows UK (http://www.ceh.ac.uk/data/nrfa/peakflow_overview.html) and against the monthly maxima available at CEH Wallingford, to ensure that the identified peaks corresponded to genuine high flows. Water‐years in which less than 90% of the flow data were recorded were discarded from the analysis, to ensure that no potentially large event would be missing from the analyzed data sets.

Catchment averaged daily rainfall series for both catchments were extracted from a national grid of daily rainfall totals at a 1 km resolution obtained by interpolating the observed values of a dense gauging network [*Keller et al*., 2005]. In the years for which the peak flow data were available for the catchments under study, the national network had approximately between 3000 and 5000 functioning gauges. To give a representation of the potential for high rainfall in each year and season the 99th percentile of the daily rainfall series for each year and season were used for each catchment. In a national scale study, *Prosdocimi et al*. [2014] had found that the 99th percentile of the annual catchment averaged daily rainfall series was significantly correlated to block maxima values for most catchments in the UK.

Finally, for the Lostock at Littlewood Bridge catchment, yearly URBEXT values are constructed by interpolating between the decadal URBEXT point estimates.

## 4 Methods

Identifying the effect of urbanization on extreme events using block maxima and point process models requires the extraction of two different data sets. The complete record of instantaneous flow recorded in a period of *M* years at a gauging station consists of n* flow measurements recorded every 15 minutes, $r=(r_{1},\ldots ,r_{n^{*}})$. The corresponding annual maxima (AMAX) series is denoted as $q=(q_{1},\ldots ,q_{M})$ and is formed by selecting the single maximum value recorded in each water‐year. Also, seasonal maxima series can be extracted by considering the maximum flow recorded in the summer (April–September) and winter (October–March) months. Conversely, peaks‐over‐threshold (POT) data consist of a series of independent events extracted from the original ***r*** record by selecting only independent events exceeding a certain high threshold value, denoted *u*. If a total of *n* threshold exceedances are extracted from ***r***, the corresponding POT series is denoted $y=(y_{1},\ldots ,y_{n})$. In this study, the procedures presented by *Bayliss and Jones* [1993] were used to ensure independence between the extracted threshold exceedances. Rather than the classical POT model, this study uses the more general point process characterization for POT data [*Smith*, 1989; *Katz et al*., 2002], which allows for a more direct modeling of covariate effects on both the frequency and the magnitude of threshold exceedances simultaneously.

The selection of the threshold to be used when building a POT series is a nontrivial task, and a number of tools exist to select sensible threshold values [*Coles*, 2001; *Lang et al*., 1999]. This selection is even more complicated when it is unsure whether the underlying series is nonstationary: the nonstationarity in the flow series could be reflected in the use of a threshold changing with the covariates influencing the original flow series, as discussed in *Kyselý et al*. [2010]. In order to facilitate the comparison of results across the two different catchments and across the annual or seasonal divisions the threshold *u* was selected to be the value for which an average of four events per year (annual series) or two events per season (winter and summer series) are recorded. The final POT annual series are also largely comparable to the series obtained following the standard practice in the UK of choosing a threshold such that an average of five independent events per year are kept in a POT series [*Bayliss and Jones*, 1993]. The chosen threshold levels have a return period of about 1.2 years, and identify relatively high peak flows.

Different modeling strategies will be deployed to investigate the effect of urbanization and climate variability on the magnitude of extreme events. Nonstationary GEV models (section 4.1) are used for the annual and seasonal maxima series, and point processes (section 4.2) are used for the annual and seasonal threshold exceedances.

### 4.1 Nonstationary Block Maxima

Block maxima are typically assumed to come from some heavy‐tailed distribution, such as the Generalized Extreme Value (GEV) distribution, which can be shown to be the limiting distribution of maxima [*Coles*, 2001]. Assuming that *Q*, the random variable describing flow maxima, follows a GEV distribution, the pdf and cdf of *Q* are defined as [*Hosking and Wallis*, 1997]:

(1) $$f_{q}(q)=\sigma^{-1}e^{-(1-\xi )t-e^{-t}},>t=\left\{ \begin{array}{l} -\xi^{-1}\ln(1-\xi (q-\mu )/\sigma ),\;\;\text{when}\;\xi \neq 0\\ (q-\mu )/\sigma ,\;\;\text{when}\;\xi =0 \end{array}\right.,$$

(2) $$F_{q}(q)=\exp\{-e^{-t}\},$$

where *μ*, *σ*, and *ξ* are the location, scale, and shape parameters, respectively. The set of flow values *q* in which the function is defined is determined by the shape parameter *ξ* as: −∞<q≤μ+σ/ξ if *ξ* > 0; –*∞* < *q* < *∞* if *ξ* = 0; μ+σ/ξ<q<∞ if *ξ* < 0.

In the stationary case, the sample of block maxima ***q*** is assumed to come from a GEV distribution Q∼GEV(μ,σ,ξ), with all the parameters constant. In the nonstationary case, one or more of the parameters can be assumed to be changing as a function of one or more covariates. A simple way to include such dependence in the model structure is, for example, to allow the location parameter to depend linearly on some covariates (*X*_1_, …, *X_p_*) so that $\mu (X_{1},\ldots ,X_{p})=\beta_{0}+\sum_{j=1}^{p}\beta_{j}X_{j}$, where the *β_i_* values are the (*p* + 1) regression model parameters. The location of the distribution would then have a different value for each observation *i* according to the corresponding value of the observed covariates sample $x_{i}=(x_{1i},\ldots ,x_{pi})$.

The relatively short records which are typically available can undermine the capability of an analysis of AMAX data to detect relevant changes in flood patterns. The use of POT series ensures that larger samples are used in change detection. In particular, as discussed in section 5.3, the analysis of AMAX data can be influenced by specific characteristics of some years.

### 4.2. Threshold Exceedances: A Point Process Characterization

POT series contain information on two different processes: (i) the frequency at which a certain high threshold is exceeded and (ii) the magnitude of the peak flows. Typically, the number of events recorded in each year is assumed to be Poisson distributed, while the magnitude of the exceedances above the threshold *u* is assumed to be distributed according to a Generalized Pareto (GP) distribution [*Lang et al*., 1999]. It can be shown [e.g., *Coles*, 2001] that the annual maxima *Q* of a flow record in which the threshold exceeding process follows the standard Poisson‐GP assumption for POT data, are asymptotically GEV distributed: Q∼GEV(μ,σ,ξ).

Exceedances above the threshold can be considered as a random process in which information on the fact that an exceedance occurred (and therefore the total number of exceedances) and the magnitude of the exceedance itself are of interest. Rather than using two separate processes to describe the threshold exceedance rate and the magnitude of the exceedance itself, it would be advantageous to characterize the different aspects of threshold crossing simultaneously. For example, for a fixed threshold *u*, a threshold exceeding process with a heavier tail is expected to result in more exceedances of the threshold, i.e., the threshold exceedance rate should be related to the threshold value *u* and to the properties of the tail of the flow distribution. The point process characterization of threshold exceedance allows such relationship to be explicitly modeled, thus allowing for a simpler and more elegant model. See *Coles* [2001] and *Katz et al*. [2002] for a discussion of point processes and their use in the analysis of hydrological extremes.

In the theoretical development, the flow observations *r_i_* in the complete record ***r*** are assumed to be independent from each other, and to have an equal probability p=Pr{R>u} of exceeding the threshold. Even if the independence of all the *r_i_* observations does not hold, the results which follow can be shown to be valid once independent peaks are extracted from the original sample. In particular, for a fixed threshold *u*, the probability of exceeding the threshold, *p*, can be derived from reworking equation (2) as (see Appendix A):

(3) $$p=\text{Pr}\{R>u\}\approx \frac{1}{n^{*}}{\left[1-\xi \frac{\left(u-\mu \right)}{\sigma }\right]}^{1/\xi }.$$

The total number of threshold exceedances can then be described by a Binomial process $\text{Bin}(n^{*},p)$, with mean *λ* = pn*, which can be approximated by a Poisson distribution Pois(λ). For a threshold *u*, a subset of *n* independent peaks would be larger than *u*. A point process *P_n_*, which records the fact that an exceedance of the threshold *u* was observed and the value of the exceedance itself *Y_i_*, is defined as

$$P_{n}=\{(i/(n+1),Y_{i}):i=1,\ldots ,n\},$$

where the first component is a counter for the number of threshold exceedances and is standardized to the [0, 1] scale as (*i*/(*n* + 1)) to simplify the notation later on. For a given threshold *u*, the *P_n_* process contains information on the number of data points above *u* observed on the whole [0, 1] interval and the magnitudes of the threshold exceedances, which have values within [*u*,*∞*).

A point process *P*(*A*) in a subset of the plane $A=(t_{1},t_{2})\times [u,\infty )$ (with $\left(t_{1},t_{2})\subset [0,1\right]$), which spans the space between the two time points (*t*_1_, *t*_2_) in the abscissa and the space between [*u*,*∞*) in the ordinate, would record the number and magnitude of events above the threshold observed in the region *A*. Threshold exceedances are assumed to be independent from each other and equally probable in each part of the [0, 1] time line, so that the number of threshold exceedances recorded in *A* should be dependent on the value of the threshold *u* and on the properties of the threshold exceeding process, and should be proportional to the width of the interval (*t*_2_– *t*_1_). The number of events recorded in the region $A=(t_{1},t_{2})\times [u,\infty )$ is thus distributed as a Poisson with mean Λ(A):

(4) $$\Lambda (A)=\Lambda ((t_{1},t_{2})\times [u,\infty ))=(t_{2}-t_{1}){\left[1-\xi \frac{\left(u-\mu \right)}{\sigma }\right]}^{1/\xi }.$$

The point processes characterization of threshold exceedances thus allows for a unified modeling framework for both the number of exceedances above the threshold and the magnitude of such exceedances. The magnitude and number of exceedances are strictly connected: for a fixed threshold *u*, a process characterized by fatter tails (i.e., larger exceedances magnitudes) would result in a more frequent crossing of the threshold. Point processes make the modeling of such connection straightforward, since the average number of exceedances in a year, which is proportional to the equation shown in (4), is described by the parameters of a GEV distribution: *μ*, *σ*, and *ξ*.

This is a particularly useful feature when investigating nonstationarity series, as the exceedance rate can change as a function of relevant covariates in a pattern similar to the one which is observed in the exceedance magnitude. One can then model one or more of the parameters as function of some covariates (*X*_1_, …, *X_p_*). For example, the effect of some covariates (*X*_1_, …, *X_p_*) on the *μ* parameter can be investigated by fitting a model such as $\mu (X_{1},\ldots ,X_{p})=\beta_{0}+\sum_{j=1}^{p}\beta_{j}X_{j}$, so that the impact of (*X*_1_, …, *X_p_*) on both the size and frequency of flood events can be assessed simultaneously.

In this work point, processes are employed to model the annual and seasonal peaks‐over‐threshold (POT) data, and to investigate the potential changes in both the frequency and the magnitude of above the threshold events. As a matter of comparison, nonstationary block maxima models as described in section 4.1 are also investigated.

### 4.2 Summary of Models Used in the Study

Two types of data were extracted from the continuous flow record at both annual and seasonal scale for both the urban and the rural catchment:

1. The block maxima values, i.e., annual and seasonal maxima. The random variable describing these values is denoted by *Q*.
2. The values across the whole record and across the seasonal records which exceed a fixed threshold *u*, with *u* chosen differently for each of the annual and seasonal series. The threshold exceedances are extracted from the raw $\left(r_{i},\ldots ,r_{n^{*}}\right)$ data set as independent peaks. The random variable describing these values is denoted by *Y*.

For each catchment, a set of covariates (*X*_1_, …, *X_p_*) is available, providing quantitative representations of potential drivers of change and variability in the flood records. These include (i) the 99th percentile of the daily rainfall of each season or year (*rain*), (ii) the water‐year in which any event was recorded (*time*), and (iii) for catchment 70005, the URBEXT value in each year (*urbext*). The covariates available in this work are at best a rough approximation of all the different aspects which underlie the flood generation process, but they can still be useful to understand the contribution of different elements on high flows.

To assess the potential drivers of change in high flows, different models are constructed, in which the effects of the covariates on the parameters describing the flood process are quantified. Further, the estimated impact of each covariate is compared between the urban and rural catchments to verify if the effect is different in the catchment with increasing urbanization. The estimated models investigate the effect of the covariates on the location parameter *μ*, and only linear effects are considered: a visual check of the relationship between the different covariates against the response variables *Q* and *Y* does not show any striking nonlinear relationship. Models to take into account the effect of covariates on the scale or shape parameter could be evaluated within both the annual maxima and the point process modeling framework. Initial attempts to have the scale parameter changing as a function of the covariates indicated that this yields to much less significant improvements in the likelihood than considering change only in the location. Consequently, this work will only consider change in the location parameter, and the associated challenges of incorporating covariates into block maxima and point process models. Nevertheless, the modeling frameworks presented in this work could potentially be employed to investigate changes in all parameters of the distribution.

Both annual and seasonal data are analyzed to investigate if the potential changes appear to be more pronounced in any of the seasons. Since the seasonal data are a subset of the annual data, the interpretation of results for the seasonal analyses should take the results for the annual series into account.

A summary of the models used in this study is given below and in shown schematically in Table 1.

**Table 1:** Summary of the Models Fitted to the Block Maxima and Peaks‐Over‐Threshold Data

	Model	Model Name	Covariates			Location Function
			*rain*	*time*	*urbext*	
Block maxima *Q* ∼	BM(μ,σ,ξ)	BM_0_	°	°	°	*μ* = *β*_0_
	BM(μ(rain),σ,ξ)	BM_1_ *_r_*	X	°	°	$\mu (rain)=\beta_{0}+\beta_{1}rain$
	BM(μ(time),σ,ξ)	BM_1_ *_t_*	°	X	°	$\mu (time)=\beta_{0}+\beta_{2}time$
	BM(μ(rain,time),σ,ξ)	BM_2_ *_rt_*	X	X	°	$\mu (rain,time)=\beta_{0}+\beta_{1}rain+\beta_{2}time$
	BM(μ(urbext),σ,ξ)	BM_1_ *_u_*	°	°	X	$\mu (urbext)=\beta_{0}+\beta_{3}urbext$
	BM(μ(rain,urbext),σ,ξ)	BM_2_ *_ru_*	X	°	X	$\mu (rain,urbext)=\beta_{0}+\beta_{1}rain+\beta_{3}urbext$
Point process *Y* ∼	PP(μ,σ,ξ)	PP_0_	°	°	°	*μ* = *β*_0_
	PP(μ(rain),σ,ξ)	PP_1_ *_r_*	X	°	°	$\mu (rain)=\beta_{0}+\beta_{1}rain$
	PP(μ(time),σ,ξ)	PP_1_ *_t_*	°	X	°	$\mu (time)=\beta_{0}+\beta_{2}time$
	PP(μ(rain,time),σ,ξ)	PP_2_ *_rt_*	X	X	°	$\mu (rain,time)=\beta_{0}+\beta_{1}rain+\beta_{2}time$
	PP(μ(urbext),σ,ξ)	PP_1_ *_u_*	°	°	X	$\mu (urbext)=\beta_{0}+\beta_{3}urbext$
	PP(μ(rain,urbext),σ,ξ)	PP_2_ *_ru_*	X	°	X	$\mu (rain,urbext)=\beta_{0}+\beta_{1}rain+\beta_{3}urbext$

#### 4.2.1 Block Maxima Models

The following models are fitted to the block maxima (*Q*), assuming a Generalized Extreme Value distribution:

1. Model BM_0_: Q∼GEV(μ,σ,ξ) with all parameters estimated as constants—this is the stationary case.
2. Model BM_1_ *_r_*: Q∼GEV(μ(rain),σ,ξ) with the location modeled as a function of the 99th percentile of the daily rainfall, $\mu (rain)=\beta_{0}+\beta_{1}rain$. This model assesses the effect of the potential for high rainfall on the high flows recorded in each year.
3. Model BM_1_ *_t_*: Q∼GEV(μ(time),σ,ξ) with the location modeled as a function of the water‐year in which each event is recorded, $\mu (time)=\beta_{0}+\beta_{2}time$. This model corresponds to the more standard models fitted in many trend studies, and estimates the effect of time on high flows.
4. Model BM_2_ *_rt_*: Q∼GEV(μ(rain,time),σ,ξ) with the location modeled as a function of both rainfall and time $\mu (rain,time)=\beta_{0}+\beta_{1}rain+\beta_{2}time$. This model estimates the effect of each one of the two covariates given that the other covariate is also taken into account. The value of *β*_2_ represents the residual effect of time after the potential for high rainfall in each year is included in the model.

The following models are also fitted to the data from the urbanizing catchment:

1. Model BM_1_ *_u_*: Q∼GEV(μ(urbext),σ,ξ) with the location modeled as a function of the urban extent $\mu (urbext)=\beta_{0}+\beta_{3}urbext$. This model evaluates the impact of the increasing urbanization on high flows.
2. Model BM_2_ *_ru_*: Q∼GEV(μ(rain,urbext),σ,ξ) with the location modeled as $\mu (rain,urbext)=\beta_{0}+\beta_{1}rain+\beta_{3}urbext$. Similar to Model BM_2_ *_rt_*, this model assesses the effect of both covariates together.

The models BM_1_ *_u_* and BM_2_ *_ru_* are an improvement compared to the standard trend analysis in the sense that URBEXT, a variable which relates to key properties of the catchment, rather than time, is employed as covariate. Although URBEXT and time are correlated, and, for this catchment, no decrease in URBEXT is recorded in time, using URBEXT rather than time would deliver a better inference in terms of the ability to quantify the effect of changes in the catchment on the high‐flow process.

#### 4.2.2 Threshold Exceedances Models

Next, a set of point process models are defined, which use threshold exceedances to investigate the effect on extreme flows of the same covariates used for the block maxima models. These same models fitted to the block maxima are fitted to the threshold exceedances *Y*:

1. Model PP_0_: Y∼PP(μ,σ,ξ).
2. Model PP_1_ *_r_*: Y∼PP(μ(rain),σ,ξ).
3. Model PP_1_ *_t_*: Y∼PP(μ(time),σ,ξ).
4. Model PP_2_ *_rt_*: Y∼PP(μ(rain,time),σ,ξ).
5. Model PP_1_ *_u_*: Y∼PP(μ(urbext),σ,ξ).
6. Model PP_2_ *_ru_*: Y∼PP(μ(rain,urbext),σ,ξ).

When fitting all the models presented in Table 1, the values of *rain*, *time*, and *urbext* are rescaled to (0, 1) to make the estimated *β_i_* parameters comparable.

The parameters of each model are estimated using the maximum likelihood (ML) estimation procedure, which allows to build confidence intervals based on the approximate normality of ML estimates. The estimated values of the regression coefficients *β_i_* and of the scale and shape parameter *σ* and *ξ*, with the corresponding 95% confidence intervals, are computed by numerically maximizing the likelihood functions described in Appendix A.

## 5 Results

### 5.1 Block Maxima Regressions

**Figure 3:**
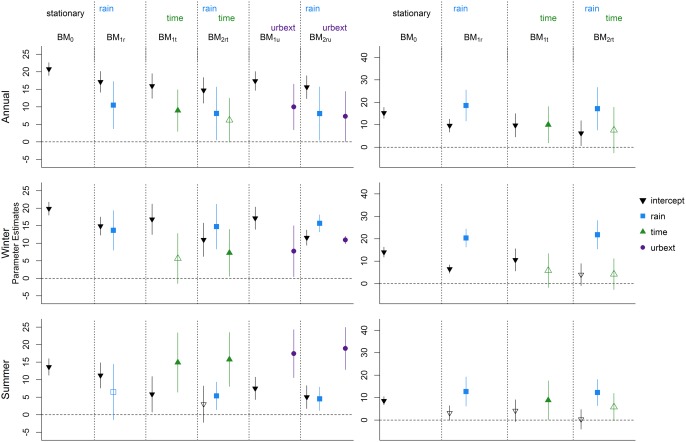
Results for the block maxima models: results for (left) the urbanized catchment and (right) the rural catchment; results for (top) the annual series, (middle) the winter series, and (bottom) the summer series.

Results for all the six GEV models (one stationary, five nonstationary) fitted to the annual maxima data for the urban catchment are presented in the top left corner of Figure 3. The difference between each model resides in the covariates used to model the location parameter, while the scale (*σ*) and shape (*ξ*) parameters are assumed to be constant and not related to the covariate values. ML estimates for *σ* and *ξ* and their standard errors in each model are shown in Table 2. The table also shows the (double negative) log‐likelihood and the Akaike Information Criterion (AIC) values for each model. These values can be used to assess the potential improvements which adding one or multiple variables can have in the model performance. As discussed in *Coles* [2001], *Galiatsatou and Prinos* [2007], and *Madsen et al*. [2014], the log likelihood values can be used to perform likelihood ratio (LR) tests and evaluate if the addition of a covariate in a model corresponds to a substantial increase in the variance explained by the model. LR tests can be performed only for nested models, i.e., models for which the model with less parameters can be obtained by constraining some of the parameters of the model with more parameters. For example, BM_1_ *_r_* is nested within BM_2_ *_rt_*, since BM_1_ *_r_* corresponds to BM_2_ *_rt_* with *β*_2_ = 0. A likelihood ratio test at a confidence level *α* is built by comparing the values of the difference between the double log likelihood of two nested models against the (1 − *α*) quantile of a $\chi_{k}^{2}$ distribution, with *k* being the difference in the number of parameters between the two models. For example, for the winter series of the rural catchment the difference of the double likelihoods of the BM_1_ *_r_* and BM_0_ models is 24.05, while it is 2.16 for a test of BM_1_ *_t_* against BM_0_: the first value is larger than 3.84 (approximately the 95th quantile of a $\chi_{1}^{2}$), which indicates that adding *rain* as a covariate significantly increases the likelihood, while the second LR test indicates that adding *time* alone as a covariate does not add much to explanatory power of the model. Similarly, one can test the significance of BM_2_ *_rt_* against BM_1_ *_r_* and BM_1_ *_t_*: the two LR test have values 2.18 and 24.07, indicating that adding *time* once *rain* is included in the model does not yield a significant increase in the likelihood. In contrast, if only *time* had been added in the model in the initial step, the addition of *rain* would highly increase the explanatory power of the model.

**Table 2:** Estimate (Standard Error) of the Scale and Shape Parameters, Negative Log Likelihood, and AIC for the GEV Models^a^

	Model	Urban Catchment				Rural Catchment			
		$\hat{\sigma }$ (s.e.)	$\hat{\xi }$ (s.e.)	−2Log‐Lik	AIC	$\hat{\sigma }$ (s.e.)	$\hat{\xi }$ (s.e.)	−2Log‐Lik	AIC
Annual	BM_0_	4.78 (0.67)	0.03 (0.13)	206.38	212.38	6.18 (0.92)	0.21 (0.16)	210.51	216.51
	BM_1_ *_r_*	4.27 (0.62)	0.01 (0.14)	199.62	207.62	4.53 (0.70)	−0.02 (0.17)	198.98	206.98
	BM_1_ *_t_*	4.07 (0.60)	−0.07 (0.13)	199.47	207.47	5.79 (0.85)	0.24 (0.15)	205.09	213.09
	BM_2_ *_rt_*	3.96 (0.57)	−0.02 (0.13)	195.94	205.94	4.82 (0.85)	0.17 (0.23)	**195.90**	**205.90**
	BM_1_ *_u_*	4.15 (0.59)	−0.03 (0.13)	199.18	207.18				
	BM_2_ *_ru_*	4.04 (0.57)	0.01 (0.12)	**195.69**	**205.69**				
Winter	BM_0_	5.03 (0.66)	0.20 (0.11)	209.36	215.36	5.55 (0.84)	0.19 (0.17)	204.26	210.26
	BM_1_ *_r_*	3.85 (0.55)	0.15 (0.14)	193.51	201.51	2.96 (0.54)	−0.26 (0.21)	180.21	188.21
	BM_1_ *_t_*	4.86 (0.62)	0.19 (0.09)	207.00	215.00	5.24 (0.77)	0.15 (0.15)	202.10	210.10
	BM_2_ *_rt_*	4.10 (0.71)	0.37 (0.22)	189.54	199.54	3.19 (0.64)	−0.07 (0.29)	**178.03**	**188.03**
	BM_1_ *_u_*	4.74 (0.61)	0.19 (0.09)	205.32	213.32				
	BM_2_ *_ru_*	4.86 (1.16)	0.85 (0.29)	**182.42**	**192.42**				
Summer	BM_0_	5.54 (0.95)	−0.08 (0.21)	220.38	226.38	4.83 (0.68)	−0.05 (0.12)	216.52	222.52
	BM_1_ *_r_*	5.57 (0.88)	0.01 (0.18)	217.30	225.30	3.89 (0.54)	0.00 (0.12)	199.14	207.14
	BM_1_ *_t_*	4.52 (0.72)	−0.16 (0.16)	209.96	217.96	4.86 (0.67)	0.06 (0.12)	212.15	220.15
	BM_2_ *_rt_*	4.02 (0.70)	−0.24 (0.19)	205.02	215.02	3.79 (0.50)	0.05 (0.11)	**195.62**	**205.62**
	BM_1_ *_u_*	4.12 (0.73)	−0.27 (0.20)	207.81	215.81				
	BM_2_ *_ru_*	3.66 (0.70)	−0.35 (0.23)	**202.56**	**212.56**				

a Bold values indicate the lowest negative log likelihood and AIC attained.

Comparing the log likelihoods of nested models via LR tests allows for a formal testing procedure, although this is only valid for nested models. To compare models which are not nested, and rank models fitted to the same data set the Akaike Information Criterion (AIC) [*Akaike*, 1973] can be used. The AIC is a measure that is also based on the log likelihood value attained by each model. Higher values of likelihood are obtained when adding more parameters in a model, so the AIC is constructed by subtracting to the log likelihood a penalty component equal to the number of parameters used in each model. For a model parametrized by *p* parameters, a log likelihood value $\text{log}‐\text{lik}(\hat{M})$ is computed and the AIC is typically defined as $\text{AIC}=-2(\text{log}‐\text{lik}(\hat{M})-p)$. Models which fit the data very well but have a large number of parameters are penalized over models which might yield a similar log likelihood value using a smaller number of parameters. Models with lower AIC should be preferred to models with higher AIC, but unlike the LR test, no cutoff value is given to determine whether the difference between two AIC values is large enough to dismiss one model. To allow for a full comparison between all models, both the log likelihood and the AIC values are reported in Table 2, while detailed information on the estimation of the location functions are presented in Figure 3.

In Figure 3, estimates for the regression parameters *β_i_* in the location models are indicated by the colored symbols, with each color and symbol identifying a specific covariate. The colored bars represent the 95% confidence intervals for the parameters. The first location model, the stationary case BM_0_, has a constant location *β*_0_, and its estimate is shown as a black downward triangle ( ▼) and is located in the top left of the plot. The second model (BM_1_ *_r_*) includes the 99*^th^* annual rainfall quantile as a covariate and the estimated *β*_1_ value and confidence interval are shown as a blue square ( ▪) and line. The symbols and lines in this second model indicate the estimated values and 95% confidence intervals for both *β*_0_ and *β*_1_ in model BM_1_ *_r_*, respectively. Similarly, estimates of *β*_0_ and *β*_2_ for the model with time as the only covariate (model BM_1_ *_t_*) are shown in the third block of the plot as a black downward triangle and a green upward triangle ( ▲). The same symbol and color scheme applies for the estimates of models in which both the 99th rainfall quantile and time are used to model the location (BM_2_ *_rt_*). Finally, estimates for the urbanization parameter (*β*_3_ in model BM_1_ *_u_* and BM_2_ *_ru_*) are shown as purple dots ( •). The horizontal dashed line which indicates the 0 value is drawn and if a confidence bar crosses the dashed line, the parameter cannot be considered significantly different from 0 at a 95% confidence level and is shown as a hollow symbol.

Overall, Figure 3 summarizes the results for all six GEV regression models fitted to the block maxima of all seasons for both the urbanized and the rural catchment. For each plot, the symbol and color scheme discussed above was used, except that results for the rural catchment (right plots) never include urban extent as a covariate. Noticeably, time appears to have a significant effect in the annual and summer series of the rural catchment when time only is included in the model (BM_1_ *_t_*), but falls just short of being significant if rainfall is also included in the model (BM_2_ *_rt_*) for the summer series. The effect of rainfall in the summer series of the urban catchment is not significant when only rainfall is included in the model (BM_1_ *_r_*) and is less markedly significant than in the other seasons when time or urbanization enter the model. This is partially due to the influence of a particular high‐flow event recorded in 1983, as discussed in section 5.3. The effect of urbanization appears to be markedly significant for the annual and the summer series, while in the winter series it is almost nonsignificant; see section 5.3 for further discussion. The likelihood ratio tests which can be built using the information in Table 2 can also be used to understand the impact of including each covariate in the regression model. For the annual series of the urban catchment, for example, a LR test of BM_2_ *_rt_* against BM_1_ *_r_* has a value of 3.68 and falls very short of being significant, while when the urban extent is included in the model (BM_2_ *_ru_*) the LR test against BM_1_ *_r_* with a value of 3.93 is just about significant at a 95% confidence level. The BM_2_ *_ru_* model also attains the lowest AIC value, an additional indication that this would be the preferred model for the data under study.

### 5.2 Point Processes

**Figure 4:**
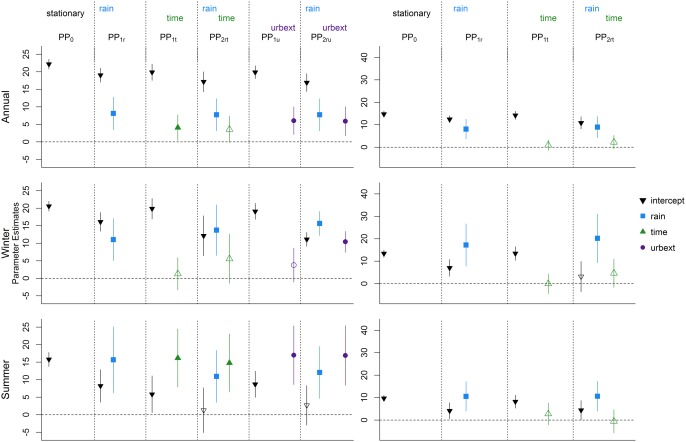
Results for the point process models: results for (left) the urbanized catchment and (right) the rural catchment; results for (top) the annual series, (middle) the winter series, and (bottom) the summer series.

Results for all six point process models for all seasons (annual, summer, and winter) in both the urbanized and the rural catchment are presented in Figure 4, using the same symbols and color scheme as in Figure 3. Results for the scale and shape parameters, along with the negative log likelihoods and the AIC values, are shown in Table 3. One first notable feature of the results is that, unlike the results for the block maxima, for all catchments and seasons, rainfall is a significant covariate. Once rainfall is taken into account (PP_2_ *_ru_*), the urbanization extent appears to be significant for all seasons, with a very strong signal appearing in the summer series. If only urbanization is included in the model (PP_1_ *_u_*) for the winter series, it appears to be nonsignificant, but it is a nonnegligible covariate when rainfall is included (PP_2_ *_ru_*). This shows that including the rainfall information can lead to a different understanding of the net impact of urbanization. Also, while urbanization is significant in the PP_2_ *_ru_* model, time is not significant in PP_2_ *_rt_*, which indicates that the increase observed in the winter high flows is not constant, but changes at a speed related to the increase of impervious cover in each year. This shows the advantage of describing the changes in the high flows generating process as a function of a covariate which describes the actual changes in the catchment rather than looking at changes on the temporal scale only.

**Table 3:** Estimate (Standard Error) of the Scale and Shape Parameters, Negative Log Likelihood, and AIC for the Point Process Models^a^

	Model	Urban Catchment				Rural Catchment			
		$\hat{\sigma }$ (s.e.)	$\hat{\xi }$ (s.e.)	−2Log‐Lik	AIC	$\hat{\sigma }$ (s.e.)	$\hat{\xi }$ (s.e.)	−2Log‐Lik	AIC
Annual	PP_0_	4.57 (0.39)	0.06 (0.08)	569.29	575.29	4.49 (0.58)	−0.23 (0.13)	519.18	525.18
	PP_1_ *_r_*	4.45 (0.34)	0.12 (0.07)	554.16	562.16	4.20 (0.40)	−0.02 (0.09)	495.62	503.62
	PP_1_ *_t_*	4.59 (0.40)	0.05 (0.08)	564.15	572.15	4.48 (0.57)	−0.22 (0.13)	518.52	526.52
	PP_2_ *_rt_*	4.46 (0.35)	0.11 (0.07)	550.59	560.59	4.18 (0.39)	0.00 (0.09)	**493.32**	**503.32**
	PP_1_ *_u_*	4.57 (0.40)	0.05 (0.08)	559.65	567.65				
	PP_2_ *_ru_*	4.45 (0.35)	0.11 (0.07)	**546.10**	**556.10**				
Winter	PP_0_	4.49 (0.55)	0.11 (0.14)	373.39	379.39	4.60 (0.70)	−0.07 (0.20)	354.51	360.51
	PP_1_ *_r_*	4.56 (0.46)	0.28 (0.09)	356.83	364.83	4.95 (0.55)	0.20 (0.10)	334.07	342.07
	PP_1_ *_t_*	4.48 (0.55)	0.11 (0.14)	373.09	381.09	4.60 (0.70)	−0.07 (0.21)	354.51	362.51
	PP_2_ *_rt_*	4.66 (0.46)	0.41 (0.16)	353.84	363.84	4.98 (0.54)	0.24 (0.10)	**331.90**	**341.90**
	PP_1_ *_u_*	4.46 (0.54)	0.10 (0.14)	371.05	379.05				
	PP_2_ *_ru_*	4.65 (0.45)	0.67 (0.17)	**346.66**	**356.66**				
Summer	PP_0_	6.57 (0.76)	0.15 (0.09)	410.34	416.34	4.32 (0.57)	−0.06 (0.13)	379.34	385.34
	PP_1_ *_r_*	6.63 (0.71)	0.25 (0.10)	393.95	401.95	4.54 (0.54)	0.09 (0.10)	361.18	**369.18**
	PP_1_ *_t_*	6.35 (0.74)	0.07 (0.08)	390.86	398.86	4.41 (0.57)	0.00 (0.12)	377.85	385.85
	PP_2_ *_rt_*	6.31 (0.71)	0.11 (0.08)	380.05	390.05	4.52 (0.54)	0.08 (0.11)	**361.14**	371.14
	PP_1_ *_u_*	6.37 (0.73)	0.09 (0.08)	389.77	397.77				
	PP_2_ *_ru_*	6.32 (0.71)	0.12 (0.08)	**376.65**	**386.65**				

a Bold values indicate the lowest negative log likelihood and AIC attained.

For the rural catchment, time is never a significant covariate and no changes can be detected for the high flows of this catchment in any season. The AIC values for the PP_2*rt*_ models in all seasons are very close to the PP_1_ *_r_*, indicating that the additional complexity in the model obtained by adding one variable is not compensated by a noticeable increase in the likelihood. For the summer season in fact, the lowest AIC is attained by the PP_1_ *_r_* model. The fact that no significant effect of time is detected in the rural catchment, combined with the strong significance of the *urbext* parameters in the urban catchment gives evidence of a significant effect of the increased urbanization levels on the location parameter of the distribution of peak flows. Compared to the results for the block maxima shown in Figure 3, the assessment of the statistical significance of the covariates differs. In particular, differences are seen in the significance of the rainfall variable in the rural catchment and the effect of rainfall and urbanization on the winter series in the urban catchment, where a strong link between change in floods and change in urbanization is identified.

### 5.3 The Effect of Influential Points

**Figure 5:**
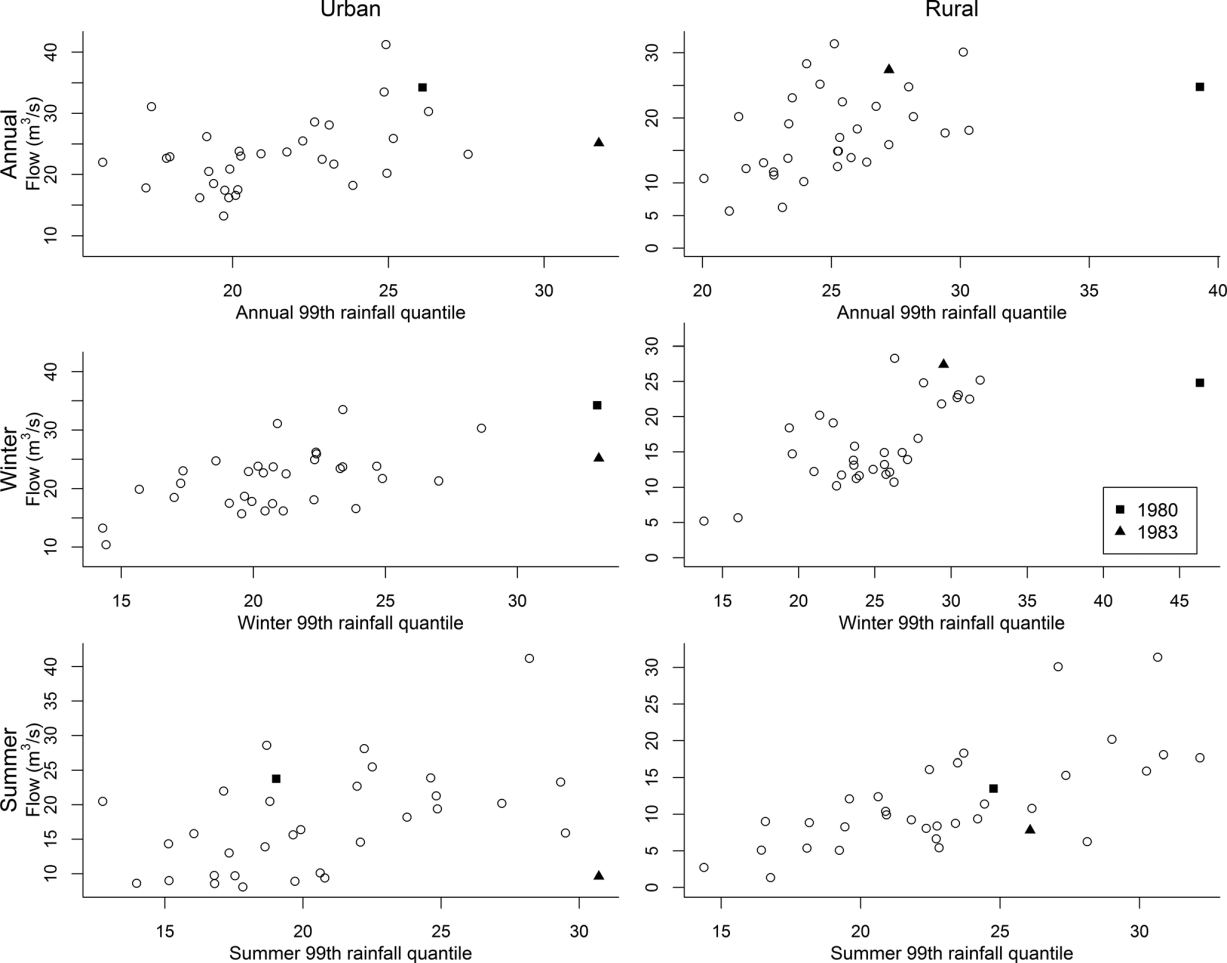
Scatterplot of annual and seasonal maxima against the appropriate rainfall covariate. Data points for the year 1980 and 1983 are indicated, respectively, as squares and triangles.

The exceptional events which characterize some years can have a large influence in the assessment of significance of the different covariates. In Figure 5, the annual and seasonal maxima series for each catchment are plotted against the corresponding 99th rainfall quantile of the catchment averaged daily rainfall. The values corresponding to the events in 1980 and 1983 are indicated as, respectively, squares and triangles. Visually, it would appear that for some series the events in these years are leverage points. Notably for the urbanized catchment the event in 1983 is characterized by very high potential rainfall values, although the maximum flow in this year is not equally extreme; the summer flow maximum recorded in this year is very low. The events recorded in year 1980 were characterized by very high winter 99th rainfall percentiles for both catchments and very high annual 99th rainfall percentile for the rural catchment. The recorded values for the annual and winter flow maxima in this year are fairly high and in line with the general shape of the relationship between the rainfall variable and flow maxima. For the urbanized catchment, the odd behavior of the 1983 data point can partially be explained by the fact that, although in 1983 very high values were recorded for the 99th rainfall quantile (31.75 mm), the year was not particularly wet and was characterized by an average daily rainfall of 2.68 mm, in line with the overall average daily rainfall of 2.76 mm. On the other hand, the high 99th rainfall quantile value of 1980 coincided with a fairly wet year with a mean daily rainfall well above the average (3.73 mm).

**Figure 6:**
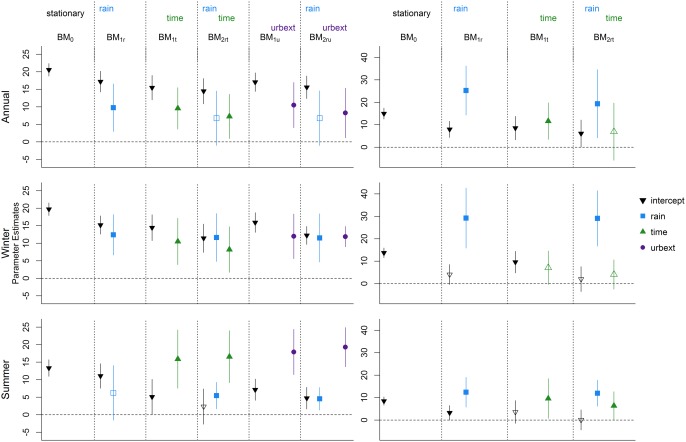
Results for the block maxima models for series without the data point of 1980: results for (left) the urbanized catchment and (right) the rural catchment; results for (top) the annual series, (middle) the winter series, and (bottom) the summer series.

**Figure 7:**
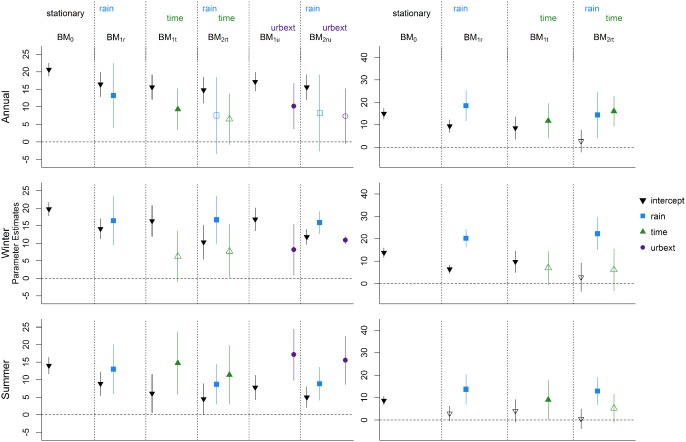
Results for the block maxima models for series without the data point of 1983: results for (left) the urbanized catchment and (right) the rural catchment; results for (top) the annual series, (middle) the winter series, and (bottom) the summer series.

In Figures 6 and 7, the results for the GEV models fitted to the block maxima without the data points of 1980 and 1983, respectively, are shown. These should be compared with the results shown in Figure 3. Unsurprisingly, the biggest differences between the results for the complete series and the results of the modified series can be seen for the catchments and seasons for which either the data point of 1980 or the data point of 1983 was visibly different from the bulk of the data points. For example, for the winter series of the urbanized catchment, a more pronounced effect of time and urbanization is visible in Figure 6. The 1980 winter record is characterized by a high rainfall and a high‐flow value. In contrast, the 1983 winter, is characterized by a rainfall value of magnitude similar to the one of 1980, but by a much smaller flow value. Since both records are also characterized by relatively low URBEXT values, the difference in the flow value cannot be explained by this additional covariate in the models fitted to the whole data set. When the 1980 event is removed, the relatively modest peak flow of 1983 in the presence of a high rainfall can partially be explained by the low URBEXT value recorded in that year.

Considering the urban catchment, removing the 1980 annual, winter, or summer events from the data set lowers the estimated effect of the rainfall variable, while the estimated effect of urbanization increases. For the rural catchment, the removal of the 1980 leverage point has the opposite effect and allows the estimated effect of rainfall to increase. A similar effect is observed for the summer series for the urbanized catchment when the data point for 1983 is removed: the estimated effects of rainfall in the left bottom corner of Figure 7 are stronger than the ones seen in Figure 3. This is due to the relatively low flow maxima registered in the summer of 1983 despite the rainfall variable being one of the highest on records. Removing the 1983 event also changes the significance assessment of the rainfall variable in the BM_2_ *_rt_* and BM_2_ *_ru_* models in the urbanized catchment.

The interpretation of the results is not radically changed if the year 1980 or 1983 is removed from the data set, but the strength and the significance of some results is slightly different. The differences in the results for the point process models (not shown) when the data for year 1980 or 1983 are similar to the ones seen for the GEV model, although somewhat smaller, since more data points are used to fit the model and the parameters show less variability. This stresses once more the challenges connected with attribution of change in block maxima series: due to the relative short series it is enough for one point to behave somehow differently from the main pattern for the results to become so variable that they can mask the actual signal of change. The use of POT data ensures that larger sample sizes are used for trend detection, making the testing procedure generally less variable and more powerful.

## 6 Conclusions

Overall, the results for the point process models presented in section 5 indicate that there is a statistically significant effect of increased urbanization levels on the high flows recorded at the Station 70005 for all seasons such that the magnitude and frequency of floods increase with increasing urbanization extent. This effect is significant in all seasons, with a stronger impact detected for the summer extreme flows. The observed effect has been shown to be present especially when the high year to year variability, represented by process‐related variables such as the 99th quantile of daily rainfall is taken into account in a nonstationary model. Further, no statistically significant effect of time has been detected in a paired, almost pristine, nearby catchment which is hydrologically similar to the urbanized catchment under study. Since URBEXT, a variable specific to the actual urbanization process, rather than time is used in the model, the effect identified by the statistical models can be directly attributed to the land use change from predominantly rural in 1970 to heavily urbanized by 2010.

Peaks‐over‐threshold series, rather than block maxima, have proven to be useful to perform such attribution. The use of POT data rather than block maxima results in larger samples which are representative of only the high end of the hydrograph and can be less affected by specific conditions observed in one year. In this study, the point process characterization of POT series is advocated, rather than the traditional POT approach. Point processes allow for a unique framework in which the effect of different covariates on the process parameters can be easily included. The direct inclusion of the covariates and the larger series used when analyzing threshold exceedances allow for a better assessment of the impact of urbanization on high flows.

The point processes framework has been employed to assess the impact of different covariates on high flows and to carry out flood‐frequency analysis in a nonstationary framework. Nevertheless, such analysis requires the availability of long records of the instantaneous flow data and of the covariates of interest, like a good measure of land use change and some summary information of the rainfall observed in the catchment. The high demands in term of data availability and modeling continues to make the attribution of drivers of changes in high flows a challenging task.

## Appendix A

### A1. Derivation of Equation (3)

Given a set of independent identically distributed random variables $\left(R_{1},\ldots ,R_{n^{*}}\right)$ with common distribution function *F_R_*(*x*), the distribution of $M_{n^{*}}=\text{max}(R_{1},\ldots ,R_{n^{*}})$ can be derived as

(A1) $$\text{Pr}(M_{n^{*}}\leq u)=\text{Pr}(R_{1}\leq u)\times \ldots \times \text{Pr}(R_{n^{*}}\leq u)=F_{R}{\left(u\right)}^{n^{*}}$$

by virtue of the independence of the *R_i_*.

Taking the traditional Extreme Value theory result: $F(g(M_{n^{*}}))\to GEV(\mu ,\sigma ,\xi )$, with $g(M_{n^{*}})$ an appropriate standardization of $M_{n^{*}}$, from equation (2) follows:

(A2) $$F_{R}{\left(u\right)}^{n^{*}}\approx \exp\left\{-{\left[1-\xi \frac{u-\mu }{\sigma }\right]}^{1/\xi }\right\}.$$

It then follows that

(A3) $$n^{*}\ln F_{R}(u)\approx -{\left[1-\xi \frac{u-\mu }{\sigma }\right]}^{1/\xi }.$$

Using a Taylor expansion of $\ln F_{R}(u)$ around *F_R_*(*u*) = 1 gives

(A4) $$\ln F_{R}(u)\approx -\left\{1-F_{R}(u)\right\}.$$

which, combined with equations (A2) and (A3), gives

$$\text{Pr}(R>u)=1-F_{R}(u)\approx -\ln F_{R}(u)\approx \frac{1}{n^{*}}{\left[1-\xi \frac{u-\mu }{\sigma }\right]}^{1/\xi }$$

### A2. Likelihood Function for a (Nonstationary) GEV Model

Denote by $q=(q_{1},\ldots ,q_{M})$ the vector of *M* observed block maxima. The log likelihood to be maximized to derive ML estimates for the *μ*, *σ* and *ξ* parameters of a GEV distribution with ξ≠0 can be derived from equation (1) as

(A5) $$\begin{array}{c} l(\mu ,\sigma ,\xi ;q)=\sum_{i=1}^{M}\ln(f(\mu ,\sigma ,\xi ;q_{i}))\\ \;\;\;\;\;\;\;\;\;\;\;\;\;\;\;\;\;\;\;\;\;\;\;\;\;\;=-M\ln\sigma -\sum_{i=1}^{n}\left\{t_{i}(1-\xi )+e^{-t_{i}}\right\} \end{array}$$

taking $t_{i}=-\xi^{-1}\ln(1-\xi (q_{i}-\mu )/\sigma )$.

For the nonstationary case in which the location is defined as a function changing linearly with one covariate *X*, i.e., $\mu (x)=\beta_{0}+\beta_{1}$, the log likelihood would then become a function to be maximized over four parameters (*β*_0_, *β*_1_, *σ*, and *ξ*), and is obtained by conveniently adjusting (A5) as

$$l(\beta_{0},\beta_{1},\sigma ,\xi ;q,x)=-M\ln\sigma -\sum_{i=1}^{n}\left\{t_{i}(1-\xi )+e^{-t_{i}}\right\}$$

taking $t_{i}=-\xi^{-1}\ln(1-\xi (q_{i}-\beta_{0}-\beta_{1}x_{i})/\sigma )$.

### A3. Likelihood Function for a (Nonstationary) Point Process Model

The likelihood for a point process model can be derived from the threshold exceedance process building on the Generalized Pareto assumption for the threshold exceedances.

Consider that out of the *n** observations $\left(r_{1},\ldots ,r_{n^{*}}\right)$, only a small number of independent peaks *n* exceeds the threshold *u*, while for $\left(n^{*}-n\right)$ observations the only information relevant to the extremal part of the distribution is that they are below the threshold. Denoting as *Y* the random variable which describes the magnitude of the peaks above the threshold, the likelihood of the threshold exceedance model can then be written as

(A6) $$\begin{array}{c} L(\mu ,\sigma ,\xi ;r)=\underset{r_{i}\;\text{under}\;\text{the}\;\text{threshold}}{\underset{︸}{\prod_{i=1}^{n^{*}-n}\text{Pr}(r_{i}<u)}}\;\;\underset{r_{i}\;\text{peaks}\;\text{above}\;\text{the}\;\text{threshold}}{\underset{︸}{\prod_{i=1}^{n}\{\text{Pr}(Y_{i}=y_{i})\}}}\\ \;\;\;\;\;\;\;\;\;\;\;\;\;\;\;\;\;\;\;\;\;\;\;\;\;\;\;={\left(\text{Pr}(R<u)\right)}^{n^{*}-n}\prod_{i=1}^{n}\text{Pr}\{Y_{i}=y_{i}\}. \end{array}$$

Points not exceeding the threshold contribute to the first component. The nonexceedance of the threshold happens with probability 1 − *p*, with *p* defined in (3). The first component can then be further reworked to be

(A7) $$\begin{array}{c} {\left(\text{Pr}(R<u)\right)}^{n^{*}-n}\approx {\left(1-p\right)}^{n^{*}}\approx \exp\{-n^{*}p\}\\ \;\;\;\;\;\;\;\;\;\;\;\;\;\;\;\;\;\;\;\;\;\;\;\;\;\;\;\;\;\;\;\;\;=\exp\left\{-{\left[1-\xi \frac{\left(u-\mu \right)}{\sigma }\right]}^{1/\xi }\right\}, \end{array}$$

where the fact that *n* is small compared to *n** and that *n** is large are used.

The second component of the likelihood, which describes the contribution of the actual threshold exceedance, assuming a Generalized Pareto distribution ( $Y∼GP(\tilde{\sigma },\xi )$, with $\tilde{\sigma }=\sigma +\xi (u-\mu )$), can be reworked to be

(A8) $$\begin{array}{c} \text{Pr}\{Y_{i}=y_{i}\}=\text{Pr}\{Y_{i}>u\}\text{Pr}\{Y_{i}=y_{i}|Y_{i}>u\}=pf(y_{i}-u;\tilde{\sigma },\xi )\\ =p{\tilde{\sigma }}^{-1}{\left[1-\frac{\xi (y_{i}-u)}{\tilde{\sigma }}\right]}^{-1+1/\xi }\\ \;\;\;\;\;\;\;\;\;\;\;\;\;\;\;\;\;\;\;\;\;\;\;\;\;\;\;\;\;={\left(n^{*}\right)}^{-1}{\left[1-\xi \frac{\left(u-\mu \right)}{\sigma }\right]}^{1/\xi }{\tilde{\sigma }}^{-1}{\left[1-\frac{\xi (y_{i}-u)}{\tilde{\sigma }}\right]}^{-1+1/\xi }.\\ \;\;\;\;\;\;={\left(\sigma n^{*}\right)}^{-1}{\left[1-\xi \frac{\left(y_{i}-\mu \right)}{\sigma }\right]}^{-1+1/\xi } \end{array}$$

Plugging the results of equations (A7) and (A8) in (A6) gives the likelihood of a point process:

(A9) $$L(\mu ,\sigma ,\xi ;r)\propto \exp\left\{-{\left[1-\xi \frac{\left(u-\mu \right)}{\sigma }\right]}^{1/\xi }\right\}\sigma^{-1}\prod_{i=1}^{n}{\left[1-\xi \frac{\left(y_{i}-\mu \right)}{\sigma }\right]}^{-1+1/\xi }.$$

For the nonstationary case in which the location parameter is taken to be a linear function of the covariate *X*, $\mu_{i}=\beta_{0}+\beta_{1}x_{i}$, the likelihood in equation (A9) becomes

$$L(\beta_{0},\beta_{1},\sigma ,\xi ;r,x)\propto \sigma^{-1}\prod_{i=1}^{n}\exp\left\{-{\left[1-\xi \frac{u-\beta_{0}-\beta_{1}x_{i}}{\sigma }\right]}^{1/\xi }\right\}{\left[1-\xi \frac{y_{i}-\beta_{0}-\beta_{1}x_{i}}{\sigma }\right]}^{-1+1/\xi }.$$
